# Magnetically Compatible Brain Electrode Arrays Based on Single-Walled Carbon Nanotubes for Long-Term Implantation

**DOI:** 10.3390/nano14030240

**Published:** 2024-01-23

**Authors:** Jie Xia, Fan Zhang, Luxi Zhang, Zhen Cao, Shurong Dong, Shaomin Zhang, Jikui Luo, Guodong Zhou

**Affiliations:** 1 College of Information Science and Electronic Engineering, Zhejiang University, Hangzhou 310027, China; jiexia@zju.edu.cn (J.X.); zhangluxi@zju.edu.cn (L.Z.); jackluo@zju.edu.cn (J.L.); 2 Nanhu Brain-Computer Interface Institute, Hangzhou 311121, China; 3 The Key Laboratory of Biomedical Engineering of Ministry of Education, Qiushi Academy for Advanced Studies, Zhejiang University, Hangzhou 310027, China; fanz@zju.edu.cn (F.Z.); shaomin@zju.edu.cn (S.Z.); 4 College of Integrated Circuits, Zhejiang University, Hangzhou 311200, China; gdzhou@zju.edu.cn

**Keywords:** carbon-based biosensors, brain electrodes, SWCNTs

## Abstract

Advancements in brain–machine interfaces and neurological treatments urgently require the development of improved brain electrodes applied for long-term implantation, where traditional and polymer options face challenges like size, tissue damage, and signal quality. Carbon nanotubes are emerging as a promising alternative, combining excellent electronic properties and biocompatibility, which ensure better neuron coupling and stable signal acquisition. In this study, a new flexible brain electrode array based on 99.99% purity of single-walled carbon nanotubes (SWCNTs) was developed, which has 30 um × 40 um size, about 5.1 kΩ impedance, and 14.01 dB signal-to-noise ratio (SNR). The long-term implantation experiment in vivo in mice shows the proposed brain electrode can maintain stable LFP signal acquisition over 12 weeks while still achieving an SNR of 3.52 dB. The histological analysis results show that SWCNT-based brain electrodes induced minimal tissue damage and showed significantly reduced glial cell responses compared to platinum wire electrodes. Long-term stability comes from SWCNT’s biocompatibility and chemical inertness, the electrode’s flexible and fine structure. Furthermore, the new brain electrode array can function effectively during 7-Tesla magnetic resonance imaging, enabling the collection of local field potential and even epileptic discharges during the magnetic scan. This study provides a comprehensive study of carbon nanotubes as invasive brain electrodes, providing a new path to address the challenge of long-term brain electrode implantation.

## 1. Introduction

Brain electrophysiology serves as a pivotal tool for investigating the functionality and activity of the nervous system [1], playing a crucial role in advancing the fields of neuroscience [2], brain–computer interface technology [3], and the diagnosis and treatment of neurological disorders [4], which often require long-term implantation of brain electrodes [5]. Brain electrodes with extended implantation periods hold promise in addressing a spectrum of neurological conditions, including epilepsy [6,7], Parkinson’s disease [8], depression [9], and spinal cord injuries. A prominent example is deep brain stimulation (DBS), which can mitigate seizures, ameliorate symptoms of Parkinson’s disease, and effectively manage various neurological disorders through the enduring implantation of brain electrodes. The required implantation durations for the brain electrodes can vary widely from several months to many years [10,11]. Conventional metal electrodes frequently encounter challenges due to their easy to corrode, poor biocompatibility, and substantial stimulation-induced damage they can cause [12]. The implantation of metal brain electrodes can also trigger immune responses characterized by inflammation and the infiltration of immune cells [13]. Presently, the field still confronts the issues and limitations related to extended implantation periods, highlighting the need for the development of new material-based brain electrodes. Carbon nanotubes (CNTs), especially single-walled CNTs (SWCNTs), are characterized by their nanoscale tubular structure composed of carbon atoms. They have garnered substantial interest from researchers due to their outstanding electronic properties and biocompatibility [14,15,16,17], thus offering potential applications in brain electrodes [18]. Reports show that brain electrode arrays based on CNTs demonstrate improved neuronal coupling, reduced stimulation-induced damage, and enhanced signal acquisition stability and accuracy [19]. The biocompatibility and stability of CNT-based brain–computer interface devices facilitate prolonged neural signal recording, thus opening up new possibilities for the advancement of brain-controlled technologies [20,21,22]. These superior qualities of carbon nanotubes offer a new approach to surmount the challenges associated with biocompatibility, immune responses, electrode performance stability, and infection during long-term brain implantation [23,24].

Meanwhile, functional magnetic resonance imaging (fMRI) is an incredibly versatile tool for studying the brain. It provides detailed images that aid in understanding brain structure, function, connectivity, and abnormalities. When combined with local field potential (LFP) acquisition by brain electrode arrays, it enables more accurate observation and localization of neural activity, thereby revealing the functional properties of various regions within the brain, which is essential for uncovering the synergistic work and information transfer between different brain regions. However, there has been limited evaluation of the behavior of brain electrodes under these conditions. The fMRI needs a magnetically compatible brain electrode array, which is difficult because the electrodes can be heated by eddy induced by magnetic field during the MRI scanning, which will hurt brain cells and introduce artifacts into the fMRI images [25]. Zhao et al. used graphene-encapsulated copper microwires as fMRI-compatible neural electrodes, which were unable to construct multiple channels due to their curved surfaces, and the copper has a magnetically compatible filament with large eddy currents, a powerful heating ramp-up, and significant artifacts [26].

In this study, a brain electrode array constructed with SWCNTs of 99.99% purity was proposed to achieve 5.1 kΩ impedance and 14.01 dB signal-to-noise ratio (SNR). This design enables the enduring implantation of brain electrodes, ensuring 3.52 dB SNR signal acquisition over 12 weeks. Furthermore, the proposed brain electrode array, operating at 7-Tesla magnetic resonance imaging, maintains a temperature change of ±0.1 °C without causing any thermal damage to brain tissue. Furthermore, the electrode exhibits no nuclear magnetic pseudo-influence on brain tissue imaging. This electrode array successfully allows simultaneous collection of electrophysiological signals and hemodynamic models. At the same time, there were fewer microglia and astroglia at the wound site of the electrodes after 12 weeks of long-term implantation compared with conventional platinum wire brain electrodes.

## 2. Materials and Methods

### 2.1. SWCNT-Based Electrode Array Fabrication

**Figure 1:**
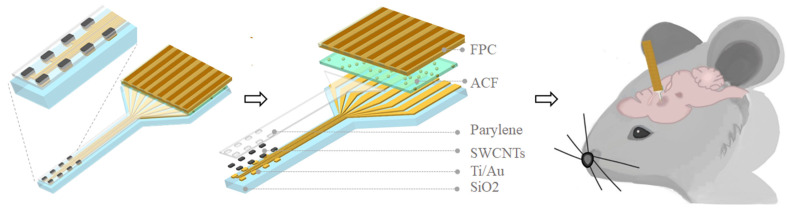
Schematic illustrations of SWCNT-based electrode array.

The proposed SWCNT-based electrode array structure is shown in Figure 1; the left is the enlarged view of the 8-channel electrodes, and the right is the layer-by-layer view. As to fabrication, the Ti/Au (80 nm) layer was deposited through e-beam evaporation and patterned by photolithography on 100 μm thickness flexible silicon wafer-coated SiO_2_. The thickness of SiO_2_ is 300 nm. Then SWCNTs with 99.99% purity were coated and patterned by inductively coupled plasma (ICP) etching. Then, the Parylene layer with 3 um thickness was deposited by evaporation as an encapsulation layer. In Supplement Text S1, we discussed the safety of SWCNTs when they were bent on Si sheets with a thickness of 100 um. The electrodes were then connected to the flexible printed circuit (FPC) using anisotropic conductive film (ACF) tape (AC-7813KM-25), which was pressed under 3 MPa at 180 degrees Celsius for 10 s. The use of flexible FPC at the interface makes the electrode have a convenient and universal interface with the post-equipment and can be compatible with a variety of acquisition equipment. The flexible FPC at the interface enables the electrode to establish a convenient and universal interface with the post-equipment, making it compatible with a variety of acquisition equipment.

**Figure 2:**
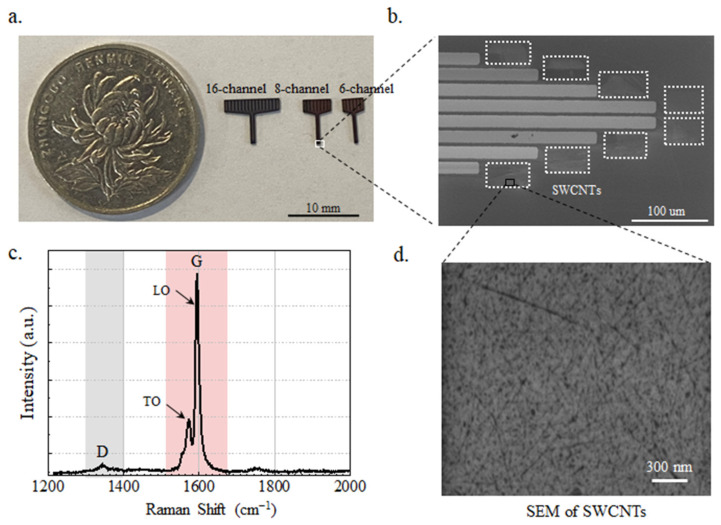
(**a**) Photos of SWCNT-based brain electrode arrays with 16, 8, and 6 channels. (**b**) SEM image of the front-end electrode. The dashed squares are the SWCNT areas. (**c**) Raman shift curve of SWCNTs. (**d**) Morphology of SWCNTs on the front-end electrode.

The fabricated SWCNT-based electrode arrays are shown in Figure 2a. The channels from left to right are 16, 8, and 6 channels; the values for the width of the front end of the probe are 395 um, 245 um, and 178 um; and the thickness of the probe is 103.383 um. Figure 2b shows an enlarged detail electrode array SEM image of the front-end electrode with each size 30 × 40 um by using a Hitachi SU5000 scanning electron microscope (SEM scanning parameter: ETH = 1.00 kV, WD = 5.3 mm, Mag = 350 X). The coated SWCNTs have a diameter of 1.0–1.4 nm and a length of 20 nm (SEM scanning parameter: ETH = 1.00 kV, WD = 4.0 mm, Mag = 30.00 K X), as shown in Figure 2d. Figure 2c presents the Raman sweep pattern of the SWCNTs with two peaks at 1344 cm^−1^ and 1594 cm^−1^ with LabRAM Odyssey (HORIBA TECHNO SERVICE Co., Ltd., Kyoto, Japan). The peak around 1300 cm^−1^ (ID peak) usually stands for C–H (carbon–hydrogen) stretching vibration, while the peak around 1600 cm^−1^ (IG peak) usually represents the C=C (carbon–carbon double bond) vibration, which is the vibration pattern associated with the double bond between carbon atoms. The ID/IG ratio can provide information about the ordered (graphite) and disordered (amorphous or defective) parts of a material’s structure. The ID/IG of the samples with higher crystallinity is smaller, while the ID/IG of the samples with lower crystallinity or more defects is larger. In Figure 2c, the intensity ratio of ID and IG peaks was 0.058, indicating good integrity and performance of SWCNTs [27]. LO (longitudinal optical) at 1590 cm^−1^ refers to the axial, and TO (transverse optical) at 1574 cm^−1^ to the circumferential displacement of the atoms. Experimental results on SWCNTs show that the TO phonon frequency for nanotubes with diameters of 1.1–1.4 nm [28].

### 2.2. In Vitro Recording

The impedance of the electrode was measured in a three-electrode system by a CHI760e impedance meter at 0.5–2000 Hz in 0.9% normal saline to simulate the body fluid environment in the mouse brain. The neural signal simulator (SKX-8000C, Xuzhou Mingsheng electronic Technology Co., LTD, Xuzhou, China) was used as a signal generator to generate sine wave signals ranging from 0.1 to 1000 Hz. The SWCNT-based brain electrode array was used to record electrophysiological signals in vitro in saline solution (0.9%). The brain electrode array was connected to a preamplifier (Plexon Inc., Dallas, TX, USA) before being connected to the Omniplex^®^ data acquisition equipment (Plexon Inc., Dallas, TX, USA).

### 2.3. In Vivo Experiment

#### 2.3.1. Animal Handling

Male C57BL/6J mice of 6–8 weeks old were maintained with ad libitum food and water in standard animal rooms with a controlled temperature of 22 ± 1 °C. The mice were allowed to acclimatize to the recording chambers for at least 3 days before experiments and signal recording. All experiments conformed to the People’s Republic of China Experimental Animal Management and Use Guidelines and the Guidelines for the Care and Use of Laboratory Animals of Zhejiang University. Additionally, all protocols were sanctioned by the Institutional Animal Care Committee.

The mice were administered atropine via intraperitoneal injection (IP, 0.12 mg/100 g body weight) and subsequently were applied isoflurane inhalant anesthesia (3–4% for induction and 1–3% for maintenance). Prior to the procedure, local anesthetic lidocaine was used at the incision site. Following this, an incision was made on the scalp to expose the cranial bone, which was then cleansed with a hydrogen peroxide (H_2_O_2_) solution. Mannitol injection was administered as a preventive measure to mitigate the risk of edema before craniotomy. A skull nail served as a reference point following drilling, and a craniotomy was performed to create a window measuring 1 × 1 mm^2^ in the left or right hemisphere, as necessary. A three-dimensional micro-drive screw was employed for the precise implantation of electrodes and their positional adjustments. At the center of the window where the skull was removed, electrodes were implanted 2 mm deep into the brain. To induce epileptic-form activity in the mice’s brains, penicillin (2 × 10^6^ IU/kg) was administered via intraperitoneal injection (IP).

To observe the immune response after brain electrode implantation, we employed tyramide signal amplification (TSA) polychromatic immunofluorescence staining. After surgery and long-term neural signal acquisition, the mice were euthanized by an overdose of isoflurane, followed by intracardiac injection of 4% paraformaldehyde (4% PFA; pH 7.2) through an extracorporeal perfusion pump. Subsequently, the brain was carefully extracted from the skull and submerged in a solution containing 4% paraformaldehyde (4% PFA; pH 7.2). It was then stored at 4 °C for 3–4 days. Subsequently, the brains were then covered with paraffin, frozen in a cabin at a temperature of −20 °C, and then sectioned into 2 μm coronal slices. These tissue slices were fixed after water washing, hematoxylin staining, hydrochloric ethanol differentiated, and staining. We used the microglial marker Iba-1 (red) and the astrocyte marker GFAP (green) for the observation. The fluorescence results were recorded and observed by an Olympus bx63 upright fluorescence microscope (Olympus, Westborough, MA, USA) (10× objective; DAPI, FITC, and TR|TC fluorescent filter cubes). Further analysis was performed using ImageJ software. The implantation sites of electrodes in mice and the slicing method are shown in Supplement Figure S1.

A healthy adult cat (2.5 kg, 2.5 years old, female, cultivated by Hangzhou Qizhen Experimental Animal Technology Co., Ltd., Hangzhou, China) was used as an fMRI experimental animal. Nembutal anesthesia was employed. The cat was anesthetized intraperitoneally with 35 mg/kg of 1–2% pentobarbital sodium for electrode implantation, then maintained at surgical levels of anesthesia with sodium pentobarbital (15 mg/kg) for 3 h during the surgery. SWCNT electrode was placed in the A1 cortex of the cat’s brain, and implantation depth was 2 mm by controlling a small animal stereotaxator. Aseptic technique was used throughout the procedure. After the experiment, the cat was sacrificed by perfusing it through the heart with bromformal solution.

#### 2.3.2. Neural Recordings

All neural recordings were conducted in the unipolar mode at 1000 Hz. Then, the LFP in the frequency range of 0.1–50 Hz was extracted through band-pass filtering in MATLAB (The MathWorks, Inc., Natick, MA, USA). This filtering process removed the 49.9–50.1 Hz power frequency interference using a notch filter. As a result, we obtained relatively clean and high-quality LFP data. The computation of time-frequency maps allows us to observe how the frequency components of neural signals change over time, providing valuable insights into the dynamic properties of neural activity and cognitive processes. To achieve this, we employed the short-time Fourier transform (STFT) to calculate the time–frequency plot of the neural signals, enabling us to visualize the distribution of the signal in both the time and frequency domains. Finally, we calculated the signal-to-noise (SNR) to validate the signal quality obtained by the electrode. SNR is defined as the ratio of the power spectral density (PSD) of the signal to the power of the noise.

#### 2.3.3. 7-Tesla fMRI Imaging and LFP Collection

A magnetic field of 7-Tesla was used for T1-weighted structural imaging using turbo spin echo (TSE) sequence with the following parameters: TR = 2530 ms, TE = 18 ms, voxel size: 0.5 × 0.5 × 1.0 mm^3^. Since common LFP acquisition devices cannot work with ultra-strong fMRI, we have chosen a standard commercial LFP acquisition device. Specifically, we selected TDT System 3, a magnetic-compatible LFP acquisition device by Tucker-Davis technologies, with a sampling rate of 1000 Hz. Supplement Text S2 shows the schematic diagram and process of simultaneous acquisition of fMRI and LFP signals.

During MRI, a rapidly changing gradient magnetic field induces eddy currents on the surface of a substance with free electrons, which, in turn, generates heat. In order to ensure the safety requirements of MRI during scanning, the temperature of the electrode in direct contact with the human body must not be too high. To monitor the temperature change of the electrode under a high Tesla magnetic field, we implanted the electrode in the melon and detected the electrode temperature change under the influence of the magnetic field through a fiber optic temperature probe that is unaffected by the nuclear magnetic signal (Oemplus 4 ch, fiber optic temperature probe TS5, Weidmann—optocon, Germany). The melon will not produce its own temperature difference and introduce temperature error, and the temperature detection fiber can be fixed near the electrode. The optical fiber temperature probe was placed near the electrode implanted in the melon, and the optical temperature measuring device transmitted the optical signal with temperature information out of the nuclear magnetic chamber for temperature value output.

In addition, gradient magnetic fields induce currents in electrodes and wires, resulting in gradient artifacts. These artifacts typically have amplitudes exceeding 1000 times that of the LFP signal, and their frequency bands overlap with those of the LFP signal. As a result, these large-scale artifacts can overwhelm the LFP signal and are difficult to be removed directly by simple filtering. To mitigate the gradient artifacts, we employed the LOGDAE algorithm [25,29].

## 3. Results and Discussion

### 3.1. Validation In Vitro

**Figure 3:**
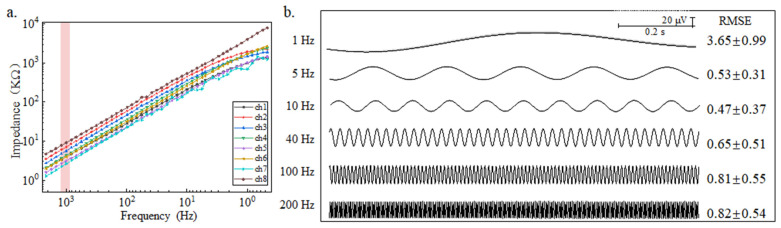
(**a**) Impedance as a function of frequency of SWCNT electrode in saline solution (0.9%). (**b**) The sine wave signal was collected by SWCNT electrode from 1–200 Hz in 0.9% normal saline.

Figure 3a shows the impedance of the SWCNT electrode in normal saline. At 1000 Hz, the impedance is 5.1 ± 1.9 kΩ, meeting the 600 kΩ impedance requirements for brain electrodes [30]. Variations in impedance between channels may arise from various wire lengths, wire thickness, and the ACF tape used for wire bonding. Corresponding to other studies, full-polymer electrodes with better biocompatibility but less stability have impedance values ranging from 7.4 kΩ [31](with 400 um × 10.5 um contact area) to 19.5 kΩ [32] (with 223 um × 223 um contact area) at 1 kHz. The low-impedance SWCNT-based electrode array can collect better signal quality. Moreover, low impedance contributes to the long-term stability of brain electrodes. The electrode array’s signal acquisition capability was assessed using artificial signals to evaluate its recording performance at an acquisition frequency of 1000 Hz. All electrode channels demonstrated excellent ability to record generated signals, exhibiting stability throughout the analysis period. Figure 3b presents the signal collected by the SWCNT electrode from 1–200 Hz, which is generated by a physiological signal generator. The signal collected by the SWCNT electrode exhibits a quality equivalent to that of the reference signal.

### 3.2. Validation In Vivo

#### 3.2.1. Detection of Epileptic Signals

**Figure 4:**
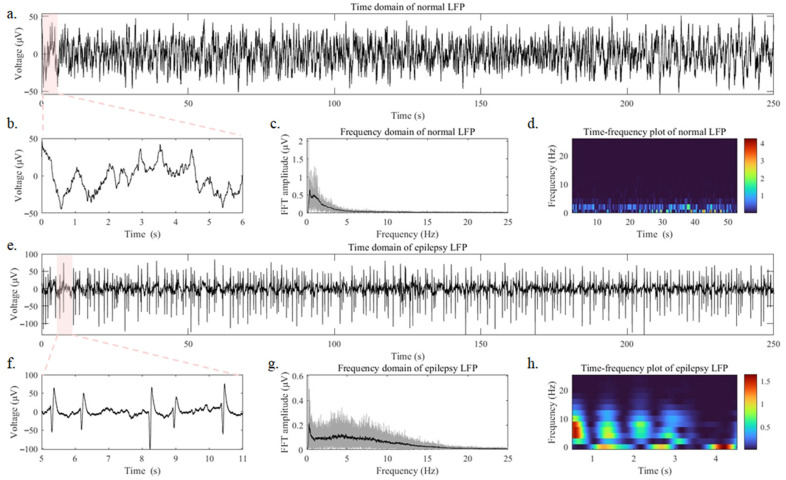
(**a**) The full-time domain diagram, (**b**) 0–6 s time domain diagram, (**c**) Frequency domain diagram, (**d**) Time–frequency diagram (0–50 s) of LFP collected by the SWCNT-based brain electrode array in a mouse under normal anesthesia. (**e**) The full-time domain diagram, (**f**) 5–11 s time domain diagram, (**g**) Frequency domain diagram, (**h**) Time–frequency diagram (0–50 s) of LFP collected by the SWCNT-based brain electrode array in a mouse under epileptic state.

To evaluate the effectiveness of the SWCNT-based electrode array when implanted into the brain, we conducted neural recordings in the right hippocampal region of mice. To investigate the electrodes’ capability to detect epileptic discharges, we induced epileptic activity in the mice by IP administration of penicillin (2 × 106 IU/kg) and compared the neural signals before and after the induction. Figure 4a displays the LFP signals recorded over 250 s under normal operation of the anesthetized mouse brain, while Figure 4b provides a close-up view of the LFP signals from 0 to 6 s in Figure 4a. No abnormal epileptic discharge signal was observed in the LFP recording of the mouse under normal conditions. In Figure 4c, the frequency domain analysis of Figure 4a reveals that LFP signals are mainly distributed within the 1–3 Hz range under normal conditions. Figure 4d, a time–frequency diagram, further illustrates that LFP signals in normal mice are mainly stable within the 1–3 Hz range over continuous time. Figure 4e depicts 250 s of LFP signals recorded during the epileptic state in the anesthetized mouse brain, while Figure 4f zooms into the details of Figure 4f, revealing intense abnormal epileptic spikes. In Figure 4g, the frequency domain analysis of Figure 4e demonstrates a rightward shift in the LFP frequency domain during epileptic states. The frequency domain distribution is mainly concentrated between 1–15 HZ, with the highest peak occurring around 5 Hz. Figure 4h provides a time–frequency diagram of 5 s, which indicates that the LFP signals in mice experiencing epilepsy exhibit continuous activity in the 1–15 Hz range, with significant variations in frequency domain distribution, and the occurrence time of epileptic events is irregular. The acquisition of LFP signals in mice effectively demonstrated the ability of the low-impedance SWCNT-based brain electrode array to collect high-quality LFP signals and detect high-frequency epileptic discharges.

#### 3.2.2. Performance of Long-Term Implantation

**Figure 5:**
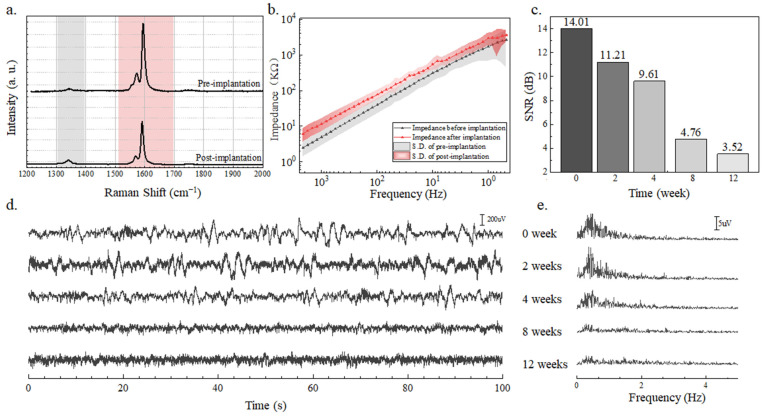
(**a**) Raman shift curve and (**b**) impedance as a function of the frequency of SWCNT brain electrode array before and after the 12-week intrusion into saline. S.D. is expressed as 8 standard deviation between channels. (**c**) The SNR, (**d**) time domain signal, (**e**) and frequency domain analysis of LFP signals recorded by the SWCNT-based brain electrode array after 0, 2, 4, 8, and 12 weeks of implantation into mice brains.

In Figure 5a, the ID/IG peak intensity ratios were 0.052 and 0.092 before and after 12-week immersion in saline, indicating that some of the carbon nanotube structures are destroyed, but the quality remains better than the standard for use [28]. Figure 5b presents the impedance curves before and after 12 weeks in saline. The impedance of the SWCNT-based brain electrode array did not exhibit significant changes, suggesting the electrode’s long-term stability. From the time domain signals (Figure 5d), we obtained the SNR of the LFP signal (Figure 5c) collected by the SWCNT-based brain electrode array implanted into mice brains for 0, 2, 4, 8, and 12 weeks. After 12 weeks of long-term implantation, effective LFP signals were successfully collected while the mice remained in good health. The longer the SWCNT-based brain electrode array was implanted into the brain, the lower the SNR of the signal because the growth of glial cells impeded the acquisition of brain signals. The SWCNT-based brain electrode array exhibits an SNR ratio of 14.1 dB, markedly outperforming traditional electrodes, like 9 dB of Pt electrode with a 1963.5 um^2^ contact area and 4 dB of Au electrode with a 1963.5 um^2^ contact area [33]. With prolonged implantation, as shown in Figure 5e, the LFP signal becomes weak due to the growth of glial cells in the brain, as well as the mice’s movement. The conclusion showed that the SWCNT-based brain electrode array had excellent long-term implantation performance.

#### 3.2.3. Long Implanted Brain Tissue Immune Response

**Figure 6:**
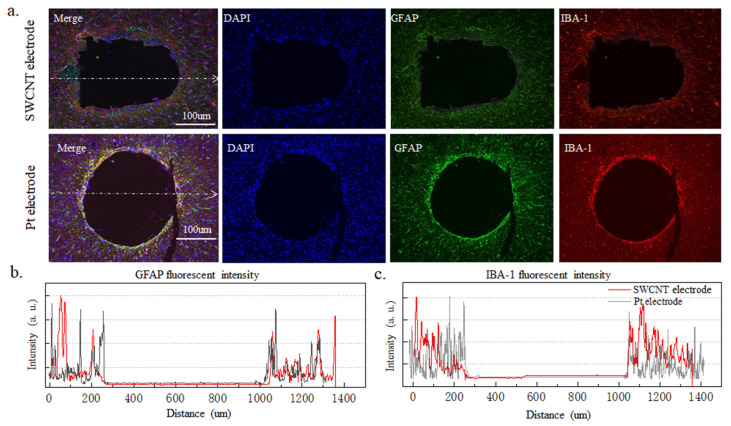
Transverse incision of brain tissue where electrodes were implanted. (**a**) The first row shows the immune results of brain cells implanted with SWCNT-based electrode arrays, and the second row shows the platinum wire electrode. (**b**,**c**) show the fluorescence intensity of Iba-1 and GFAP, respectively.

**Figure 7:**
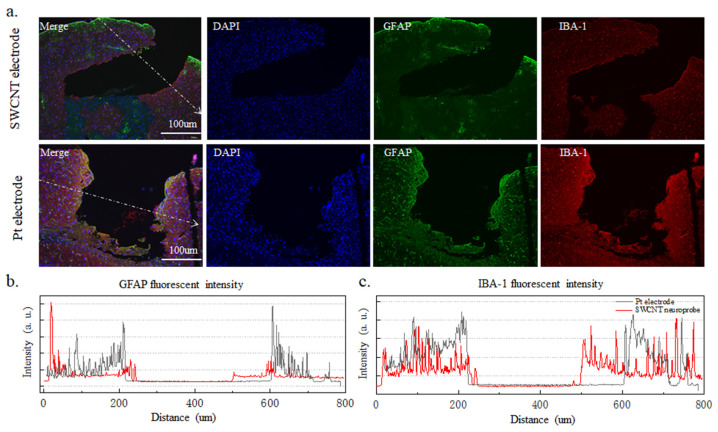
Longitudinal incisions in brain tissue where electrodes were implanted. (**a**). The first row shows the immune results of brain cells implanted with SWCNT-based electrode arrays, and the second row shows the platinum wire electrodes. (**b**,**c**) show the fluorescence intensity of GFAP and Iba-1, respectively.

The cellular immune response was characterized by the glial reaction 12 weeks after the implantation of electrodes in the cortex. Both SWCNT-based brain electrode array and platinum wire electrode with a diameter of 250 μm were implanted into the brains of mice for 12 weeks, and the immune response of brain cells was observed. In Figure 6a, the first row displays the immune response results of brain cells implanted with the SWCNT-based electrode array, while the second row shows the results of brain cells implanted with a platinum wire electrode. The immune tissue image represents a cross-section of the brain at the site of electrode implantation. In this image, the blue DAPI staining indicates the nucleus, while the red Iba-1 is a microglial marker, and the green GFAP is an astroglial marker. The growth of microglia and astrocytes around the electrode can affect the performance of the electrode and cause damage to the brain. The GFAP and Iba-1 staining images of the SWCNT-based brain electrode array show a uniform fluorescence intensity without a significant increase in the vicinity of the implantation site, as compared to the platinum wire electrode. This suggests that a SWCNT-based brain electrode array implanted for 12 weeks induced fewer astrocytes and microglia, indicating better biocompatibility. In Figure 6b,c, the fluorescence intensity of GFAP and Iba-1 was analyzed using by ImageJ software. Under the same magnification, the image pixel intensity was normalized. The fluorescence intensity of GFAP and Iba-1 around the tissues implanted with SWCNT-based brain electrode arrays exhibit less variation than that of the platinum wire electrode. Staining of the longitudinal sections of the brain with implanted electrodes in Figure 7a shows that the flexible SWCNT-based brain electrode array implantation results in smaller wounds and lower fluorescence intensities of GFAP and Iba-1 at the wound sites, which were evenly distributed. In contrast, the rigid platinum wire electrodes produced larger wounds, with higher fluorescence intensity of GFAP and Iba-1 at the wound sites, producing more astrocytes and microglia, as shown in Figure 7b,c.

It can be concluded that the SWCNT-based brain electrode array exhibits good biocompatibility, and the tissues near the implanted electrode show good healing, indicating that the SWCNT brain electrode array could be implanted into the brain to collect effective LFP for a long time. Compared with platinum wire electrodes, the SWCNT-based brain electrode array shows fewer microglia and astrocytes.

#### 3.2.4. 7-Tesla fMRI Compatibility

**Figure 8:**
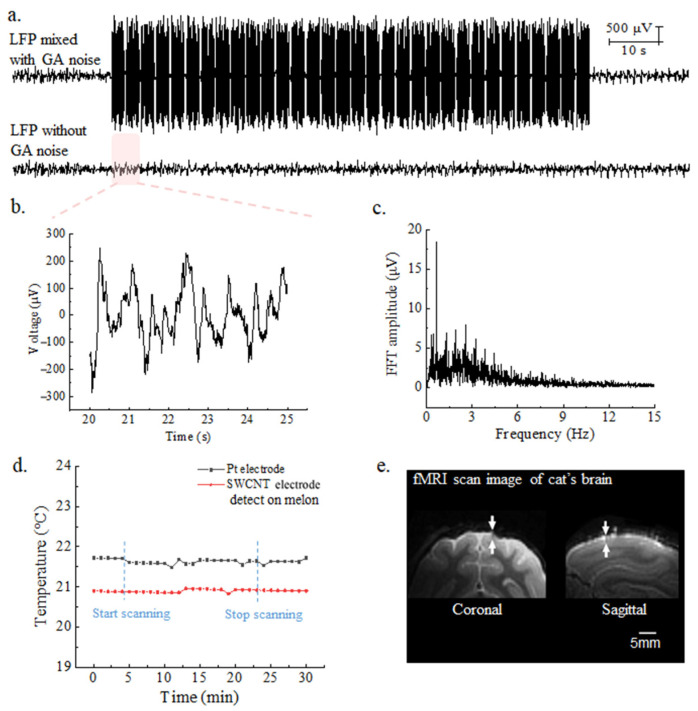
(**a**) LFP signal during EPI scanning, and Real LFP signal with GA noise removed (**b**) The LFP details after removing gradient artifacts from 20 to 25 s in (**a**). (**c**) The spectrum of epileptic LFP signal after removal of interference. (**d**) The temperature of the CNT brain electrode array and Pt electrode in melon when operating at 7-Tesla magnetic resonance imaging. (**e**) fMRI images of CNT brain electrode array implanted into the animal brain.

The SWCNT-based brain electrode array demonstrates effective functionality during 7-Tesla magnetic resonance imaging (MRI). As shown in Figure 8a, the cat’s epileptic signals (with obvious spines) were collected during EPI sequence scanning by 7-Tesla fMRI equipment. Each volume in the EPI sequence contains 32 slices. Below is the real LFP signal after removing the GA interference by the LOGDAE algorithm [25]. Figure 8b shows the LFP details after removing gradient artifacts from 20 to 25 s in Figure 8a. The frequency domain spectrum of the epileptic LFP signal after GA removal is shown in Figure 8c, which is consistent with the spectrum of epileptic LFP signal recorded from mice under a non-magnetic field condition. These results validate the findings in Section 3.2.1 conducted in vivo and demonstrate a rightward shift in the mid-frequency domain of the spectrum, confirming the accuracy and authenticity of epileptic LFP after GA removal. Figure 8d reveals that the SWCNT-based electrode array, operating within the 7-Tesla magnetic resonance imaging environment, maintains a temperature of 20.9 ± 0.1 °C on melon. In contrast, the platinum wire of the control group exhibits a temperature of 21.5 ± 0.2 °C, higher than that of the SWCNT-based electrode array. The room temperature was 21 ± 1 °C at the time of scanning. Both SWCNT-based electrodes and platinum wire electrodes do not cause large changes in temperature and can be safely implanted. Supplement Figure S2 shows an fMRI image of the melon. It is worth noting that electrodes containing magnetic materials, such as iron and nickel, could not be used within the fMRI room. The SWCNT-based brain electrode array enables the recovery of true and effective LFP signals and epileptic spines, even in the presence of nuclear magnetic interference with amplitudes thousands of times higher than the actual LFP signal. This allows for a simultaneous collection of electrophysiological signals and hemodynamic models. In order to effectively illustrate the artifact size arising from the SWCNT-based brain electrode array within brain tissue, the cat brain is chosen as an alternative over the mouse brain. This decision is based on the limitation of the mouse brain’s size, which impedes the clear demonstration of structural details. The implanted SWCNT-based brain electrode array has eight channels, with a needle tip width of 245 μm, and was implanted to a depth of 2 mm, as indicated by the white arrow in Figure 8e. The implanted electrode does not produce artifacts in the area around the electrode, allowing for high-definition fMRI images to be obtained.

## 4. Conclusions

In this work, we present a SWCNT-based brain electrode array that exhibits excellent performance in mouse models, providing a new avenue for long-term brain electrode implantation. Furthermore, the SWCNT-based brain electrode array is flexible, with good electrical conductivity and 7-Tesla nuclear magnetic compatibility. The novel flexible brain electrode based on single-walled carbon nanotubes (SWCNTs) with 99.99% purity has a low impedance of 5.1 ± 1.9 kΩ and a high signal-to-noise ratio (SNR) of 14.01 dB. The SNR remained at 3.52 dB for a 12-week period performed in mice. The superiority of carbon nanotubes as a brain electrode material can open up new opportunities for the development of neuroscience and brain–computer interface technology, offering unprecedented possibilities for the treatment and management of neurological disorders. This study provides a new pathway to overcome the challenges of long-term implantation of brain electrodes.

## Data Availability

The data presented in this study are available on request from the corresponding author. The data are not publicly available due to privacy.

## Supplementary Materials

The following supporting information can be downloaded at: https://www.mdpi.com/article/10.3390/nano14030240/s1, Supplement Text S1: The safety of SWCNTs bending on Si sheets with a thickness of 100 um; Supplement Figure S1: The implantation sites of electrodes in mice and the slicing method; Supplement Text S2: The schematic diagram and process of simultaneous acquisition of fMRI and LFP signals; Supplement Figure S2: fMRI image of the melon.
