# Supplementary material for: Magnetically Compatible Brain Electrode Arrays Based on Single-Walled Carbon Nanotubes for Long-Term Implantation
Source: Nanomaterials (Basel). 2024 Jan 23;14(3):240. doi: 10.3390/nano14030240 (PMC10856774; doi:10.3390/nano14030240)
Supplement: Supplementary file 1 [file nanomaterials-14-00240-s001.zip › nanomaterials-2803500-supplementary.pdf]

Supplement

Title：Magnetically compatible Brain Electrodes Array based on Sin-gle-walled Carbon Nanotubes for Long-term Implantation

Supplement Text S1

Too soft substrate would cause SWCNTs to break, so Si sheet with a thickness of 100 um was chosen. The base of this experiment is 100 μm thickness flexible silicon wafer-coated Sio2 with a thickness of 300 nm. Si has a Young's modulus of 168.9 Gpa and is flexible at 100 um. The Young's modulus of parylene is 2.9±0.2 GPa. The bending degree of a 4 inch silicon wafer with a thickness of 100 um(coated with SWCNTs) is shown in the figure below:

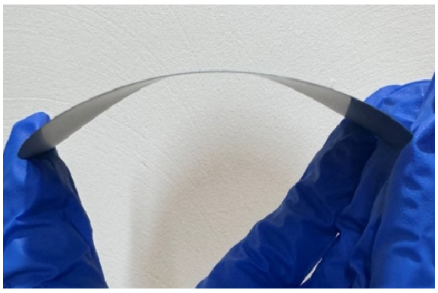

Silicon wafer bending picture

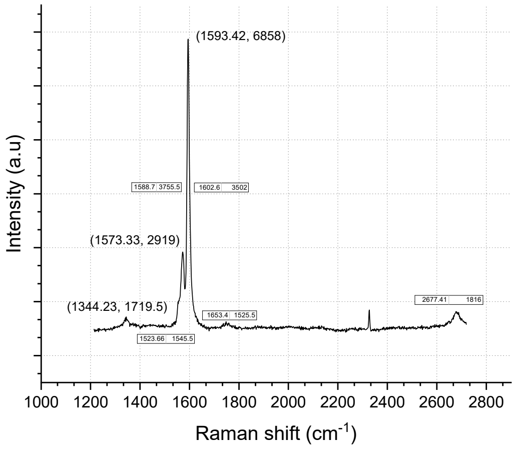

Raman shift of SWCNTs after bending

Supplement Text S2

After the electrode is implanted in the brain, it is transferred to the omnetics interface through the adapter provided by TDT to the nuclear magnetic indoor amplifier of TDT, and the signal is amplified through the amplifier and then transmitted out of the nuclear magnetic outdoor to the signal acquisition system. At the same time, 7T nuclear magnetic scans were taken to image the heads of mice.

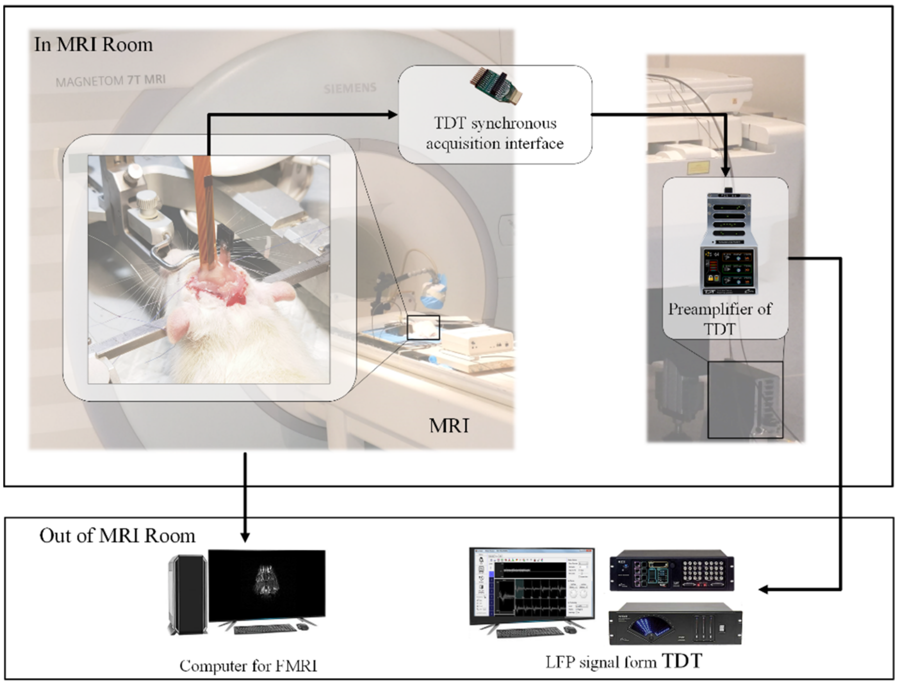

schematic diagram of simultaneous acquisition of fMRI and LFP signals

Supplement Figure S1

The implantation sites of swcnt electrode and Pt electrode as a contrast electrode in mice.

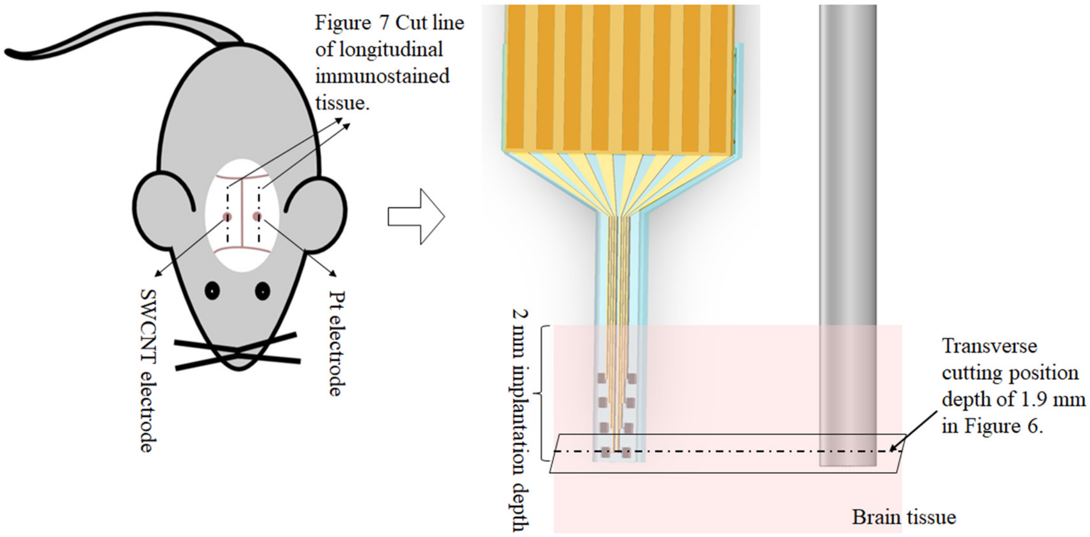

Implantation diagram for immune response

Supplement Figure S2

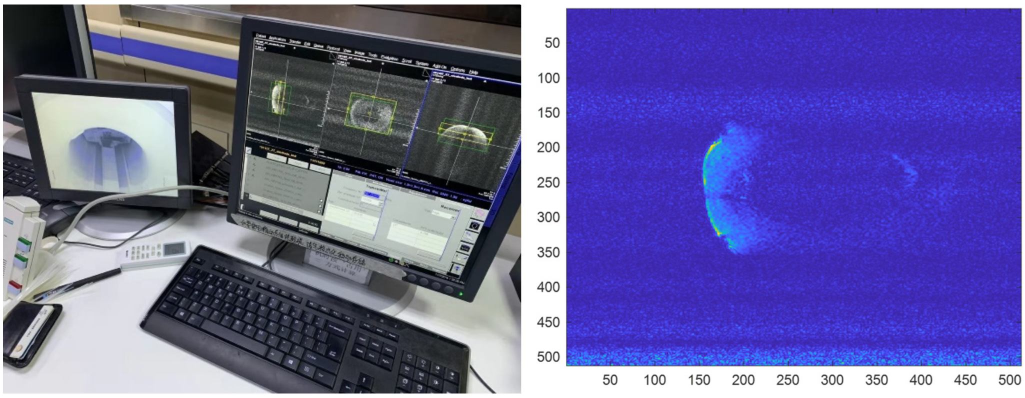

fMRI image of melon
